# Self‐responsibility, rationing and treatment decision making – managing moral narratives alongside fiscal reality in the obesity surgery clinic

**DOI:** 10.1111/hex.12651

**Published:** 2018-01-19

**Authors:** Amanda Owen‐Smith, Joanna Coast, Jenny L. Donovan

**Affiliations:** ^1^ School of Social and Community Medicine University of Bristol Bristol UK; ^2^ National Institute for Health Research Collaboration for Leadership in Applied Health Research and Care West (NIHR CLAHRC West) University Hospitals Bristol NHS Trust Bristol UK

**Keywords:** individual, morbid, obesity, qualitative, rationing, responsibility, self‐responsibility

## Abstract

**Background:**

Addressing the prevalence of severe obesity and its concomitant morbidities is widely acknowledged as one of the most pressing global health priorities. Nevertheless, a paucity of effective interventions and universal pressure on health‐care budgets means that access to obesity treatments is often limited. Although health‐care rationing can be conceived as a socially constructed process, little is known about how decisions emerge within the context of face‐to‐face doctor–patient interactions.

**Methods:**

In this study, we used in‐depth interviews and clinic observations to investigate clinicians’ (n = 11) and patients’ (n = 22) experiences of the rationing of obesity surgery and to examine how broader cultural assumptions around personal responsibility for health emerged in the context of clinical interactions.

**Results:**

Patients and clinicians worked within similar frameworks when it came to self‐responsibility for health and the appropriateness of providing publicly‐funded weight loss surgery. Issues around personal accountability dominated consultations, and patients were expected to provide narratives of the development of their obesity and to account for the failure of previous interventions. Clinicians faced the added pressure of having to prioritise a limited number of patients for surgery, which was predominantly managed through mandating pre‐referral weight loss targets.

**Discussion:**

Although clinicians sought to maintain an empathic attitude towards individual patients, in practice they were conflicted by their responsibility to ration health‐care resources and tended to rely on entrenched models of behaviour change to allocate treatment. As a result, the content of consultations was mostly focused on issues of personal responsibility, reflecting wider stigmatized attitudes towards extreme obesity.

## 1 INTRODUCTION

Clinically severe, or morbid, obesity is commonly defined as a body mass index (BMI) in excess of 40 (type III obesity), or in excess of 35 in the presence of significant co‐morbidity (type II obesity).^1^, ^2^, ^3^ Living with severe obesity is associated with a host of physical and psychiatric morbidities, most notably including diabetes, cardiovascular disorders, musculo‐skeletal complaints, depression and several cancers, and the mortality rate for this population is approximately double that for those with a healthy BMI.^4^ Effective interventions to treat severe obesity are sparse and many sets of national health‐care guidelines now recommend the use of bariatric surgery as the intervention of choice where other treatments (such as diet and exercise programmes and anti‐obesity drugs) have failed, subject to appropriate medical and psychological assessment.^1^, ^2^ Nevertheless, at least in state‐funded health‐care systems, it is widely acknowledged that access to bariatric surgery is significantly rationed, and in the United Kingdom, less than 1% of the theoretically eligible population (according to guidance from the National Institute of Health and Clinical Excellence [NICE]) are treated on the National Health Service (NHS) each year.^5^ NICE guidance states that surgery should be available to those with a BMI>40 (or>35 in the presence of significant co‐morbidity) where dieting and pharmaceutical interventions have failed to yield lasting weight loss.^1^

Rationing is frequently argued to be a ubiquitous element of health‐care provision.^6^ However, the characterization of rationing as an economic inevitability has come under question in the sociological literature, which instead highlights the social construction of the concepts of “need” and “effectiveness,” and underlines the importance of socio‐political relations in decision making.^7^ Considerations around the social construction of rationing are relevant at all levels of health‐care decision making. Even the so‐called “technical” cost‐effectiveness calculations used by organisations such as NICE in the United Kingdom are under‐pinned by political and social value judgements (such as the accord given to Quality Adjusted Life Years [QALYs]), and at the meso‐level observational studies show an ongoing “tussle” between clinicians and managerial staff for control over scarce resources with scant regard to explicit decision‐making frameworks.^8^, ^9^, ^10^ When it comes to decision making about individual patients evidence is sparser, although observations of multidisciplinary decision making have shown that moral and clinical discourses mingle as clinicians try to identify which patients have the most legitimate claim on resources.^11^, ^12^, ^13^ Interview studies with clinicians have demonstrated the stress endemic in rationing at this level, with fears of prompting distress (or anger) in patients often resulting in clinicians retreating to implicit rationing strategies whereby patients are simply not told about all the available options for treatment.^14^, ^15^

The relevance of personal responsibility as a criterion in the allocation of scarce health‐care resources has long been debated. Some ethicists have argued that the extent to which a health condition arises as a consequence of a freely chosen behaviour should impact on the degree of priority awarded to treating that individual, albeit in a rather limited way (ie the illness should not threaten life or fundamental capabilities and treatment should still be made available through a degree of copayment).^16^ There is certainly some support for this from the public with all surveys that posit the question finding majority agreement for inclusion of self‐responsibility in priority‐setting frameworks.^17^ However, more in‐depth studies with public representatives have shown that these views tend to be revised once participants are told more about the complexities of the situation and the multitude of difficulties in proving cause and effect.^18^, ^19^ Particular problems here include defining how much of an individual's health condition is caused by their behaviour or just “luck” (such as genetic make‐up or in‐utero exposures), and the amount of agency any one person might be expected to exercise over their behaviour, which is presumed to be impacted by both internal and external stressors.^20^ It is for these reasons that policymakers and clinicians alike have largely rejected the adoption of self‐responsibility into formal priority‐setting frameworks other than at a very general level.^16^ However, the extent to which personal responsibility is taken into account by clinicians in micro‐level resource allocation decisions it is unknown. In this study, we aimed to use the example of decision making in the obesity clinic to investigate the operation of rationing at the level of the doctor–patient consultation. In this article, we report how considerations around self‐responsibility for health impacted on prioritization for surgical treatment so that more can be understood about the implicit rationing mechanisms that occur when there are insufficient resources to treat all those who could potentially benefit from a treatment.

## 2 METHODS

A qualitative approach was taken to the investigation, which involved the use of interview and observations (via audio‐recording) so that both the individual views of patients and clinicians and naturally occurring interactional data could be accessed. The study took place in a UK NHS centre for obesity surgery, which saw a patient throughput of 400‐500 patients per year, all of whom were referred by primary care physicians and met NICE criteria for surgical treatment. However, only in the region of 50‐60 surgeries were undertaken each year due to funding restrictions imposed by the local NHS funding body, which could not afford to implement NICE recommendations that obesity surgery should be more widely available. A longitudinal approach was taken to research and, where possible, patients were followed up for a period of 3 years following their initial consultation (Table 1). Clinicians were interviewed once prior to any contact being made with patients.

**Table 1:** Demographic characteristics of patient informants and engagement in research

	Number of informants interviewed at each time period following their initial consultation (n = 22)					
	Recruitment(n = 22)	6 months(n = 18)	12 months(n = 12)	18 months(n = 12)	24 months(n = 12)	36 months(n = 11)
Gender						
Male	7	5	2	2	2	2
Female	15	13	10	10	10	9
Age group (at baseline)						
20‐39	9	8	3	3	3	3
40‐59	12	9	8	8	8	7
60+	1	1	1	1	1	1
Occupational group						
Professional	4	3	1	1	1	1
Other non‐manual	8	7	7	7	7	7
Manual	3	3	1	1	1	1
Not employed	6	4	2	2	2	1
Retired	1	1	1	1	1	1

A purposeful approach was taken to sampling, and all those professionals involved in the prioritisation of patients for treatment were invited to participate in a research interview. In addition, consultant physicians, who emerged as the key decision‐makers, were invited to take part in the consultation study. Patients were sampled purposefully, and anonymized records were used to identify those across a broad spectrum of ages, of both genders, and with a range of different BMIs within the morbidly obese category. Sampling and data analysis were undertaken iteratively, and initial research findings were used to guide ongoing sampling techniques.

Data collection was undertaken by AOS using in‐depth interviews and audio‐recordings of consultations. Interviews with clinicians were undertaken first, and key topics explored included general attitudes to severe obesity, the legitimacy of funding obesity surgery on the NHS and approaches taken to the prioritisation of patients for treatment. Where possible, interviews with patients were undertaken during the 2 weeks prior to their initial consultation at the obesity clinic and focussed on topics including individual illness trajectories, general attitudes to the provision of obesity treatment on the NHS, and expectations of forthcoming consultations. All interviews were audio‐recorded on a digital device, and data were transferred as soon as possible to a secure University server, usually on the same day. Where patients were in agreement, their subsequent 6‐monthly follow‐up consultations at the weight management clinic were also recorded, and further in‐depth interviews conducted (with patients only), usually within 2‐3 weeks of the consultation date. Sampling of patients continued until we had recruited patients with a reasonable spread of age, sex and BMI within the morbidly obese range. However, the emphasis was on gaining an in‐depth understanding of individual experiences, and all patients were invited to participate in each stage of data collection unless they had either specifically requested to withdraw from the research or had failed to respond to three contacts, which was interpreted as a preference not to be contacted further. It was not possible to blind the clinicians to which consultations were being recorded, as we were unable to get ethics approval for recording entire clinics.

All interviews and recordings of clinic observations were transcribed using a professional transcribing service. Initial interviews were then independently coded by all three authors and preliminary coding structures established jointly. These coding structures were then further developed by AOS, although these were frequently reviewed by JC and JD. Clinician interviews were all undertaken at the start of the study to build rapport and gain permission to observe clinics, and this data was used in the development of topic guides for patient interviews, which were coded separately. Atlas.ti was used to facilitate data management. Consultation data were coded thematically in the first instance, to establish the dominant structure of consultations and identify major themes arising therein, of which the most marked was personal responsibility for health. Individual consultations were then subjected to further scrutiny to specifically interrogate the theme of personal responsibility and link this to individual views and experiences of health‐care rationing expressed in individual interviews. Although we focused on interactional elements in analysis (such as turn‐taking, interruptions), a full Conversation Analysis approach was not undertaken.

The study was reviewed by the NHS Research Ethics Service (Wales Research Ethics Committee 3) and local NHS Research and Development bodies before any recruitment was undertaken (reference 11/WA/0020). The research was undertaken in a large county town in South West England between 2011 and 2014. Written informed consent was taken from all participants.

## 3 RESULTS

Eleven clinicians and 22 patients were recruited to the research, and in total, 78 interviews were undertaken and 22 consultations recorded over a period of 3 years. Clinician participants were primarily consultant physicians, but also included the lead dietician, the service psychologist and a specialist nurse (referred to as Allied Health Professionals [AHPs] throughout). Only one professional declined to participate in the research. All participating professionals were involved in prioritizing patients for treatment, although the consultant physicians were the key decision‐makers.

Patient participants were all new referrals, were predominantly female (15), ranged in age from 23‐60, and about half worked in professional or non‐manual occupations (12) (Table 1). All patients were white, had a BMI in the morbidly obese range, and met national expert criteria for surgical treatment. Of the 22 consultations it was possible to record, half were initial consultations, and half were follow‐up appointments. Interviews were undertaken between May 2011 and May 2014 and ranged from 40 to 90 minutes in length. Clinician interviews were all undertaken on NHS premises, and the majority of patients were interviewed at home, with two opting to be interviewed at the hospital where they were being treated, and one at the University where the researcher was based.

The results reported below relate firstly to what we have termed the overall context for decision making, where clinicians and patients were discussing personal responsibility for health and the legitimacy of public funding for obesity surgery in individual interviews, and secondly to decision making in clinic, where the impact of personal responsibility for health on clinical interactions is considered in‐depth.

### 3.1 The context for decision making

Two distinct but related themes arose when participants were discussing the overall appropriateness of obesity surgery in individual interviews, which related to (i) the excessive consumption of food, and (ii) the excessive consumption of NHS resources.

#### 3.1.1 The excessive consumption of food

Reflections on the aetiology of severe obesity were a common occurrence in interviews, and both clinicians and patients discussed this within an overall framework whereby individuals were expected to take responsibility for the health consequences of individual behaviours.

Basically, what we're trying to do is to get the patient to look at their behaviour … really thinking about the reasons why they are overweight and how they've got to that weight. (Clinician (C) 5, consultant)

I try not to lie to myself that I'm fat because I've got big bones or I don't eat enough … I know I eat too much chocolate, too much crisps. (Patient (P) 14, pre‐consultation interview)

Over‐eating was discussed as the main causative factor of severe obesity by all professional informants. However, this was almost always discussed with reference to a wider “obesogenic” environment and often with reference to a psychological basis for eating difficulties that made it difficult for particular individuals to exercise full agency over their behaviour.

We kind of think they [patients] are pretty well like alcoholics – they have an issue with food, they all know they are overweight, they all know that it is a problem with their health, they all don't want to be overweight, but they still eat the food. (C3, consultant)

For patients, accounts oscillated between an acceptance of the broader societal censure around excessive consumption and attempts to distance themselves from such judgements through explaining their actions in the context of individual life stories. The most common explanations for excessive eating included childhood socialization patterns, restrictions imposed by work or caring responsibilities, and suffering associated with physical and mental illnesses.

I spent so much time focussing on the health of my children, the health of my husband, my home, decorating I forgot about me really, and I completely neglected myself. And I put on an awful lot of weight – through stress, through lack of time, you know loads of different issues …. I was just getting bigger and bigger and bigger. (P9, pre‐consultation interview)

Further attempts to distance themselves from the negative stereotypes associated with severe obesity were made through detailed accounts of previous attempts to lose weight, which were ubiquitous across patient interviews.

There are people I know that have become very big, and they haven't really tried to lose weight and they think “oh well, I'll just get a [gastric] banding a bit later in life and I can do it without any effort that way” … [but] in my case I've not done that. I really don't want an operation and if there was an easier way of losing weight I'd be there trying to do it. (P3, post‐consultation interview)

#### 3.1.2 The excessive consumption of NHS resources

All professional informants felt the surgical treatment of severe obesity was a legitimate use of NHS resources in some circumstances. However, only one informant (C10) argued for universal and others felt there should be some conditions around its use.

It [surgery] isn't a magic answer. Erm, but for some patients it can be life transforming, no question, turn their lives round. So I think there is a place for it, but I think it needs to be [limited], you know, society needs to think very carefully about what it spends its money on…. I think that this has a small niche place but I don't think this is the solution to the obesity epidemic. (C9, consultant)

Professional accounts were clearly under‐written by a conviction that patients had to “earn” the right to surgery, and all clinicians said patients were expected to lose ten per cent of their weight at referral before they would even be considered for surgery.

Most people who are coming to us have a body mass index in the 40s and the 50s, and we have to be saying to them “come on you've got to play your part, you've got to earn this operation, because this is something that is a situation that's caused through your eating patterns”. (C4, consultant)

Patients largely agreed with clinicians that there needed to be some controls around the availability of obesity surgery, and none of those interviewed felt it should be offered to all those who were overweight. Most patients interpreted the 10% pre‐surgical weight loss target as an arbitrary figure that was a means of assessing their motivation to lose weight and take responsibility for their own health. However, this was not acceptable to the majority of informants who said they had already made multiple attempts to lose weight and were trying to access surgery as a last resort to prevent further deterioration of their co‐morbidities.

It's [weight loss target] a hard one to call. The NHS funding I think should be dependent on whether it's [condition] your own fault or if you've made an effort to change it – because if it's not your fault then somebody should help you, that only human kindness surely, but if you've made an effort to change and you really have tried and you just can't they should help you [too]. (P13, post‐consultation interview)

### 3.2 The interactional basis of health‐care delivery – decision making in clinic

The need to prioritise patients for care and the requirement that they should lose ten per cent of their bodyweight prior to being considered for surgical treatment tended to dominate initial clinic consultations. Clinicians first needed to negotiate patients’ expectations of their referral, which were usually that they had been referred for surgical assessment, and explain the clinical service on offer, which for most patients was limited to a dietetic (and sometimes psychological) intervention.

C4 I'm not going to get you ready for surgery from here. That's not how the service works. What we do is, we're the beginning, if you like.

P9 The step on the ladder.

C4 (laughs) The step on the ladder. … Some people that come here will go forward to surgery, but not everybody.

Although several clinicians openly acknowledged to patients that not all those referred into the service would eventually be treated surgically, only one (C8) related this directly to resource constraints and explained that they were trying to achieve the best overall outcomes from limited health‐care resources. This approach seemed broadly acceptable to patients, who did not contest this basis for decision making either in consultations, or in post‐consultation interviews.

C8 We want any surgery to be as safe as it can be, so we reduce the risk of chest infections and clots and all these sorts of things.

P18 Yeah.

C8 And also we only have a limited number of operations available as well and so you know we've got 400 people coming in, we've got 70 operations in [local area] so somehow we have to whittle that down and we want to find the people who are the most motivated

P18 Yeah, fine.

C8 and the most up for it and will therefore gain the most from it.

P18 Yeah, sure.

Other clinicians focused on the criteria that would secure a surgery slot for the patient at hand, which related to the attainment of a 10% weight loss target alongside satisfactory engagement with the dietetic team over a period of at least 12 months. All clinicians related this to both taking personal responsibility for health and getting the best possible health‐care outcomes for the individual patient.

If you don't control yourself with your diet and you don't have a good pattern, you still gain weight after an operation and for that reason alone we don't want to put people through a useless procedure. (C5 to P10, follow‐up consultation)

Despite many patients revealing in post‐consultation interviews that they felt distressed and frustrated at being given a weight loss target to achieve, this was never disclosed in clinic, and any contestations that were raised were related instead to the requirement that this should take a minimum length of time, so extending the wait for surgery.

### 3.3 Accounting for weight gain and recommendations for change

The majority of clinic consultation time was devoted to eliciting patients’ stories of weight gain and making recommendations for behaviour change, both of which were usually explicitly related to personal responsibility for health (Table 2).

**Table 2:** Percentage of consultation time spent in discussion of specific topics

Topics discussed	Initial consultations (average length =34 minutes)	Follow‐up consultations (average length=10 minutes)
Eating habits and lifestyle advice	34.0%	44.9%
General health check and diagnostic tests	20.5%	4.4%
Co‐morbidities	14.2%	9.3%
Local exclusion criteria and clinic process	13.4%	26.9%
Surgery	10.0%	7.0%
Other (explaining purpose of consult, patient questions, etc.)	7.5%	5.6%
Direct discussion of funding issues	0.4%	1.9%

Although the majority of clinicians clearly tried to maintain a non‐judgemental stance at the outset, patients’ contributions often became apologetic and confessional as they sought to account for their outsize bodies. Responses to these “confessions” varied with clinicians appearing at times to endorse patients’ negative self‐assessments and at others to seek to limit the emphasis on personal culpability put forward by patients. This is demonstrated by the two extracts presented in Box 1. In the first extract, the doctor attempts to comfort the patient in a number of ways. Initially, he tries to limit the culpability she accepts for her health state by alluding to “central stuff” which implies a physiological basis to her weight problem that she cannot control. He then goes on to elicit their commonalities by emphasizing their similar age and socialization experiences, which the patient engages with enthusiastically, leading to further disclosures about the aetiology of her condition. Lastly, the doctor assists her to construct a moral separation between herself and other patients, whom he denigrated as “creatures” and “less complex”, further limiting the patient's personal accountability. In extract 2, by contrast, the clinician appears to endorse patient culpability by not engaging with her initial disclosure of poor mental health and focusing instead on the behavioural aspects of her narrative. In addition, her disclosure of previous weight loss is met with little acknowledgement of success (“not bad”) and subsequently used as a platform to disclose the mandated weight target prior to consideration for surgery.

Box 1 **confessional narratives**

**Technique 1 – limiting individual culpability**

P1 You see I taught my children [to eat well], but I just didn't teach myself.

C9 Well it may be a bit more complex than that if you've always been this big all your life.

P1 Yeah.

C9 I mean there's probably some central stuff, you probably don't feel as full as other people and

P1 Mm.

C9 It's interesting because most people aren't big from childhood.

P1 No, you see, my mum was – my mum's big as well.

C9 Mm.

P1 You see it was at the end of the war wasn't it?

C9 The war, yeah, I know

P1 All the other brother and sisters were all

C9 If it's any consolation you're a year younger than me, so I kind of grew up through the same era

P1 Yeah, and you will eat what's on your plate and

C9 Seconds and all this stuff.

P1 Seconds and

C9 And don't leave the table until you've eaten it all and this stuff.

P1 And because I ate it all there was more piled on.

C9 Yeah. Okay … I think you may be a little bit more complex than some of the creatures we have to deal with

**Technique 2 – endorsing individual culpability**

P13 I've never been slim in my life, I don't know what it's like.

C2 (Pause) Any particular times when you've put on a lot of weight quickly?

P13 Um, I had no job when I was about eighteen for about eighteen months and I got very depressed, I wouldn't listen to people when they said it was best for you to get up and do things. I just sort of wallowed. I put on a lot of weight then and then I met my boyfriend … because he's very active and has got fast metabolism he doesn't put on weight, so I'd eat with him, but really I shouldn't, do you know what I mean?

C2 You wanted to match his appetite.

P13 If he was eating something I'd pick at it, but then he wasn't there I wouldn't eat that, it's okay for him because he stays slim.

C2 Okay, yeah. Um, (pause) have you [ever] lost weight?

P13 I once stayed with my mum when I was about twenty and I did lose … because she buys healthy things (laughs). Um, I did lose a little bit.

C2 How much?

P13 Maybe a stone and a half.

C2 Okay that's not bad.

P13 But, um, I always struggle to keep it off long‐term.

C2 Have you regained it?

P13 Yeah and some.

C2 It's important that you've done it because we're going to ask you to do it again.

Although some patients welcomed their clinician's advice around healthy eating, for most it repeated information they already knew, and for some, it was burdensome and patronizing, contributing to the distress they already felt around their eating patterns and body size.

If I'd heard it once I'd heard it a million times about, “ah well, you could have had an apple instead of a Kit Kat, blah, blah, blah”. … As an obese person, or an overweight person, you're faced with this all the time. And sometimes it just gets a little bit too much. (P11, post‐consultation interview)

### 3.4 Assessing progress and treatment outcomes

Nearly, the entirety of follow‐up appointments was dedicated to the discussion of patients’ success (or failure) to lose weight since their last appointment. Where patients had been successful in losing weight, this was generally met with praise and approval by clinicians, although interview data showed that this was not always interpreted in a positive light by patients.

Good girl. Oh, you're doing great. (C9 to P1, follow‐up consultation)

I just found her [AHP] incredibly patronizing. … I've lost five per cent [of weight] already … so she kept saying things like “we're all so proud of you”, “you're doing so well” … and sort of leaning right forward and I thought any minute now she's gonna hug me or something (laughs). (P21, follow‐up interview)

There were no formal reprimands of patients who had not made progress towards their weight loss targets, but patients were reminded that they were not yet eligible for surgery and there was a tacit expectation that they should provide a plan for the way forward. In two cases (P4, P5, both male), the patients clearly took on this agenda by providing detailed explanations for their failure to lose weight and giving clear assurances that this would be achieved by their next consultation, with one reinforcing to his doctor that he should not be given “e*asy targets” (P5, in consultation with C5)*. However, other (mostly female) patients took a more apologetic and submissive tone, which was normally greeted sympathetically by clinicians, several of whom had commented in initial interviews that there was no agreed plan for dealing with patients who simply failed to lose weight.

Interviewer: What happens when they [patients] don't lose their ten percent?

C2: That's a very difficult issue which I haven't worked out a way to handle. We talk about it but nobody comes up with a proper answer…. To say to somebody after two years of visits… “you're not ready for surgery, go away” … that's a very hard one.

Follow‐up interviews showed that those who continually failed to meet their weight loss targets experienced frustration at the perceived lack of support available, and some despaired as they began to regard the target, and therefore the surgery, as unattainable.

I have gained a bit of weight since [initial consultation] … I was disappointed and frustrated. … I thought maybe I was going to hear that I'm a candidate [for surgery]. … She [consultant] was telling me, “Go back to the nutritionist” (sighs). (P10, 6 month follow‐up point)

I am going through quite a bad patch and a crisis at home … I am gaining weight, I am not following any of the nutritional advice to do this and that and I have just felt desperate. (P10, 12 month follow‐up point)

I kept gaining, and am now back to about 100 [kilos]. And I truly don't know what to do. (P10, 36 month follow‐up point)

By the end of the research period, six of the 22 patients originally recruited were known to have achieved their weight loss target and accessed surgery, although only one (P2) achieved this within the projected timeline of 1 year, and others waited up to 34 months for treatment. For P2, the rapid weight loss had been accompanied by a great deal of distress and the emergence of further disordered eating patterns.

I did kind of starve myself for 4 days before the [appointment] … but if I didn't lose the weight she wouldn't have referred me. … I went to see the dietician – I wasn't honest with her really, I'd say “yeah I'll do this, do this” … but sometimes I just can't help myself … it's an addiction. (P2, 12 month follow‐up point)

Of the remaining 16 patients, 9 had disengaged from the clinical service and/or the research process, one had accessed private care, one had sought alternative treatment, and the remainder were still waiting to access NHS surgery. The protracted waiting time and requirement to lose weight before surgery was almost universally interpreted by patients in the context of NHS resource shortages.

It is all money, when it comes down to it … I think if you went private you haven't got to go through what the National Health does. (P7, 36 month follow‐up point)

## 4 DISCUSSION

Patients and clinicians worked within broadly similar frameworks when it came to the discussion of responsibility for health and the availability of publicly‐funded weight loss surgery. Both groups accepted that at least some level of personal culpability was important in the development of the condition, and this common attitude framed clinic interactions and decision‐making processes. Appointments tended to be dominated by discussions around individual narratives of weight gain and previous attempts to lose weight, which forced patients to reflect on previous deficits in their health‐related behaviour. This led to a predominance of guilt‐ridden, “confessional” narratives from patients where self‐stigmatization was rife and clinicians seemed to be accorded additional status through their habitation of healthier bodies. This gulf in status seemed to make clinicians’ other task in the consultation—to prioritise between patients for surgical treatment—easier, and patients were all told they needed to lose weight to “earn” their right to surgery. Although post‐consultation interviews showed that most patients were uncomfortable with this prioritisation technique, concerns were very rarely raised in clinic, where there was evidence of collusion between clinicians and patients to focus on behavioural aspects of obesity and ignore the evidence of wider system‐level limitations on care.

These findings offer some support to earlier observational work in obesity clinics which showed how stigmatised attitudes towards extreme overweight (on the part of both patients and doctors) shape clinical interactions and accentuate the power imbalance in the consultation.^21^, ^22^, ^23^ Nevertheless, it is notable that most clinicians in this study took a compassionate approach in consultations, and it is questionable how far the dominant behaviourist approach would have been pursued if resource availability had been more generous. Research in other clinical areas has also demonstrated the moral nature of clinical interactions, particularly when it comes to consulting for stigmatised conditions, such as gender reassignment or sexual health issues.^24^, ^25^ In addition, this data demonstrates the social construction of the rationing process whereby the standard principles for resource allocation (effectiveness and cost‐effectiveness of treatments) are supplemented when resources are extremely tight, in this case to focus on personal responsibility for health, which was assessed quantitatively through mandated weight loss targets.

This research comprises a rare attempt to observe the operation of health‐care rationing in process and supports the findings from earlier studies demonstrating the difficulties that clinicians face in being open with patients about the impact of financial limitations on care.^14^, ^15^ These earlier studies have shown that despite a commitment to being open with patients in theory, clinicians experience extreme discomfort in having to explain that there are external constraints on their ability to provide treatment and retreat to more implicit methods in practice. This pattern was clearly demonstrated here, where nearly all clinicians preferred to focus on the perceived clinical benefits of the prioritisation criteria adopted, rather than explaining that there was a shortage in the number of surgery slots available. Of particular, interest was how shared attitudes around personal responsibility for severe obesity seemed to “pave the way” for such decisions and encouraged patients to collude with implicit rationing.

The findings presented here are also relevant to broader debates around the role of personal responsibility in health‐care rationing. Certainly, this is not a novel issue, and as far back as the mid‐1990s, academic debate was extensive as to the validity of using individual lifestyle choices as a basis for rationing health care.^11^, ^16^ However, such justifications have generally been avoided in the day‐to‐day practice of health‐care priority setting, in part because of the difficulty in proving causality in any one particular case.^26^, ^27^ Nevertheless, the impact of global financial recession means resource issues are once again high on the health policy agenda, and demanding lifestyle modifications prior to publicly‐funded elective surgery is a relatively commonly reported phenomenon in both the professional and lay press.^28^, ^29^ The research reported here shows that although most patients were able to engage in lifestyle conversations when discussing their eligibility for treatment, they became frustrated if the targets appeared too challenging or they were repeatedly unable to achieve them. This information will be useful to clinicians and policymakers when considering how notions of self‐responsibility for health can be further integrated into explicit policy frameworks.^20^ Of particular importance, here is the conservation of the therapeutic relationship between doctors and patients and the need to avoid the stigmatization of marginalized groups;^16^, ^30^, ^31^ the data from this study show the difficulties patients face in openly discussing their concerns about behavioural change with clinicians, particularly when those behaviours are subject to social disapproval.

This study was strengthened by an in‐depth, ethnographic approach and the triangulation of interview and observation data in analysis. We systematically compared the accounts given by doctors and patients during in‐depth interviews to their behaviours during naturally occurring clinical interactions. Any disjunctures here (such as when clinicians expressed a preference for explicit rationing techniques in interviews, but demonstrated implicit techniques in practice) were investigated and used as triggers for deeper and more nuanced analysis. The study is limited by its focus on one clinical area and restriction to one geographic location in South West England and future research would benefit from a larger and more varied sample, alongside follow‐up interviews with clinicians to assess any changes in attitude over the study period. In addition, a key element of the data presented relates to a social acceptance of clinicians acting as gatekeepers to care, which is common in state‐funded health services. However, in largely private insurance systems, such as the US, or in state‐funded insurance systems, this expectation may be less clear‐cut, with more rationing responsibility resting with managerial staff or insurance bodies.

The research presented in this study contributes to the argument that health‐care rationing is not simply an economic, rational process, but rather a socially constructed process that emerges through a dialogue embedded in existing social relations.^7^ The broader social discourse demanding personal responsibility for health explicitly influenced decision making in clinic, but financial factors were rarely mentioned, meaning that rationing remained largely implicit and non‐accountable. The data presented here provide further insight into the operation of clinical rationing in practice and will be of use to clinicians and policymakers considering how the global rhetoric around self‐responsibility for health can be integrated into future frameworks for health‐care priority setting.
